# Evolutionary Genomics of Immunoglobulin-Encoding Loci in Vertebrates

**DOI:** 10.2174/138920212799860652

**Published:** 2012-04

**Authors:** Sabyasachi Das, Masayuki Hirano, Rea Tako, Chelsea McCallister, Nikolas Nikolaidis

**Affiliations:** 1 Department of Pathology and Laboratory Medicine, Emory Vaccine Center, School of Medicine, Emory University, USA; 2 Department of Biological Science, California State University Fullerton, USA

**Keywords:** Antibodies, gnathostomes, genomic organization, cladistic markers, microRNA, comparative genomics.

## Abstract

Immunoglobulins (or antibodies) are an essential element of the jawed vertebrate adaptive immune response system. These molecules have evolved over the past 500 million years and generated highly specialized proteins that recognize an extraordinarily large number of diverse substances, collectively known as antigens. During vertebrate evolution the diversification of the immunoglobulin-encoding loci resulted in differences in the genomic organization, gene content, and ratio of functional genes and pseudogenes. The tinkering process in the immunoglobulin-encoding loci often gave rise to lineage-specific characteristics that were formed by selection to increase species adaptation and fitness. Immunoglobulin loci and their encoded antibodies have been shaped repeatedly by contrasting evolutionary forces, either to conserve the prototypic structure and mechanism of action or to generate alternative and diversified structures and modes of function. Moreover, evolution favored the development of multiple mechanisms of primary and secondary antibody diversification, which are used by different species to effectively generate an almost infinite collection of diverse antibody types. This review summarizes our current knowledge on the genomics and evolution of the immunoglobulin-encoding loci and their protein products in jawed vertebrates.

## INTRODUCTION

All animal species from sponges to mammals have the ability to defend against infection and protect themselves from re-infection using a variety of mechanisms, which collectively are referred to as immunity. However, only vertebrates, a small fraction of the animal phylogenetic tree, have developed a specific type of immunity, the adaptive immune response. The adaptive immune system (AIS) has evolved progressively over a long period of time and in all vertebrates contains specific organs, cells, molecular cascades, and molecules [1-3].

Random evolutionary events shaped by selective forces resulted in the generation of the vertebrate AIS, which is able to discriminate between self and non-self, protect against pathogens, and allow for memory of subsequent pathogenic encounters [3-5]. The tinkering evolutionary process resulted in two types of AIS in the two major lineages of extant vertebrate species, the jawed and the jawless vertebrates [6-9]. These two radically different systems use entirely different types of molecules that are randomly assembled to generate receptor molecules on the surface of specialized cells called lymphocytes. Jawless fishes, represented by lampreys and hagfish, use the Variable Lymphocyte Receptors, in which the basic rearranging element is a leucine-rich repeat cassette, and the jawed vertebrates use immunoglobulin (Ig) receptors, in which the basic component is the immunoglobulin domain [6-9]. The use of different types of molecules represents either a case of common ancestry in which the vertebrate ancestor possessed both types of rearranging molecules or is the result of convergent evolution [7].

Immunoglobulins (Igs or antibodies) are key components of the AIS in all jawed vertebrates [3, 5], which include elasmobranches, teleosts, amphibians, reptiles, birds, and mammals. The canonical antibodies are composed of four polypeptide chains: two identical heavy (H) chains and two identical light (L) chains held together by disulfide bonds as well as extremely stable non-covalent interactions. Both the heavy and light chains are composed of a variable (V) and a constant (C) domain. The V domain is responsible for antigen-binding, whereas the C domain is responsible for binding to effector molecules, which by triggering complex signaling pathways, eliminate the antibody-coated foreign material [3, 10].

In the germline configuration, the Ig genes are composed of multiple sets of genetic elements that by recombining with each other produce the mature variable region gene that encodes the V domain. The H-chain V domain is encoded by three genetic elements: the variable-segment (V_H_), diversity-segment (D_H_), and joining-segment (J_H_) genes, whereas the light-chain variable domain is encoded by the variable-segment (V_L_) and joining-segment (J_L_) genes. The V domain can further be subdivided into two regions based on the amount of sequence divergence and structural delimitations. These regions are: the framework regions (FR) and the hypervariable or complementarity-determining regions (CDR). All functional genes encoding the V domains of both H and L chains are flanked by conserved recombination signal sequences (RSS). The consensus RSS consists of a highly conserved heptamer and less conserved AT-rich nonamer sequences separated by either 12±1 or 23±1 base-pair spacer [11-13].

The antibodies and their encoding-loci have been greatly differentiated during vertebrate evolution resulting in a diverse repertoire of Ig-encoding genes and Ig isotypes [2, 3, 10, 12-18]. The comparative and evolutionary genomics studies of the Ig-encoding loci and other immune-related genes have special significance on our understanding of the evolution and complexity of the immune system itself. Understanding how the system and its components evolved will allow us to understand how and why the immune system fails in certain individuals and what can be done to rescue these phenotypes. Such knowledge, which can be achieved only by identifying and using the information provided by the plethora of natural experiments over the course of millions of years of evolution, has the potential to contribute specific tools for vaccination, diagnosis, and treatment of several human conditions. Recently the availability of multiple draft genomic sequences from several vertebrate species has facilitated and permitted the exploration of the genomic organization and molecular evolution of several gene families that have essential roles in immunity. This review will provide a comprehensive overview of recent genomic studies of the vertebrates’ Ig-encoding loci with a focus on their mode of evolution and their evolutionary dynamics.

## IMMUNOGLOBULIN HEAVY CHAINS

### Evolution of Immunoglobulin Heavy Chain Isotypes

Cartilaginous fishes express Igs comprised of conventional heavy-light chain isotypes that are called IgM and IgD/W. Apart from these two isotypes, a third isotype called IgNAR (Ig-new antigen receptor) was found in sharks. The latter is an unusual disulphide bonded homodimeric isotype that consists of two heavy chains [19, 20]. The IgNAR isotype in contrast to the conventional antibodies, lacks Ig light chains. Such antibodies that lack light chains have also been found in camelids, but their occurrence is considered to be a lineage specific event not related to the emergence of IgNAR in sharks [21]. It has been shown that homodimeric antibody in camelids evolved from a specific lineage of V_H_ genes and have gone through a number of adaptive changes [21]. To increase the antigen-binding repertoire, the variable gene encoding homodimeric antibody has experienced higher proportions of sequence variability compared to that of variable genes of the conventional tetrameric antibodies. However, the differences in the types of antigens being recognized by tetrameric and dimeric antibodies are not clear.

The IgM and IgD isotypes are regarded as ancient isotypes that were likely present in the vertebrate ancestor predating the emergence of cartilaginous fishes. Unlike IgM, however, IgD has undergone important structural changes over the course of evolution and shows a discontinuous distribution, i.e., lost in certain birds and mammals [20, 22-24]. In addition to IgM and IgD, a third heavy-chain isotype, IgZ/IgT, has been found in bony fishes [25, 26]. The IgZ/IgT isotype, which has certain unique features, is thought to be restricted to bony fishes. The presence of both IgM and IgD isotypes in the earliest jawed vertebrates, suggests that the divergence of these two isotypes predates the separation of the bony and cartilaginous fish lineages.

One important time interval of the evolution of vertebrates is the emergence of tetrapods, which include amphibians, reptiles, birds, and mammals. Tetrapods exhibit the most diversified antibodies compared to non-tetrapod species. Placental mammals (eutheria) including humans, express five types of IgHC chains, which are denoted by the Greek letters: α (IgA), δ (IgD), ε (IgE), γ (IgG), and μ (IgM) [3] (Table **1**). In addition to these five main types, all mammals studied thus far also express variable numbers of subspecialized isotypes [27-31]. A distinct IgHC isotype has been described in platypus, termed IgO (o) [32]. The *o* gene in platypus encodes a unique IgHC isotype that consists of four constant region domains and a hinge, and is structurally different from any of the five known mammalian Ig classes. Phylogenetically, this gene is related to ε and γ, and appears to be a structural intermediate between these two genes [32]. Since the IgO isotype has thus far not been observed in any other tetrapod species, it is plausible that this isotype is restricted in prototherian mammals.

Birds possess three different H chain isotypes, IgM, IgA, and IgY (Table **1**). Among the three different classes, the IgM and IgA are homologous to the corresponding mammalian chains. The third class of antibody, the IgY, although has some similarities with both IgG and IgE of mammals does not have a homolog in the latter species. However, the IgY class has been described in both reptiles and amphibians [33-37].

Reptiles have four H-chain isotypes, μ, υ, δ, α, and four corresponding classes IgM, IgY, IgD, and IgA (Table **1**). More recently, genomic organization studies showed that the IgH locus of the Asian lizard (*Eublepharis macularius*) contains genes that code for IgM, IgD, IgY, and IgA [38-40]. In contrast to the Asian lizard, the IgH locus of the American lizard (*Anolis carolinensis*) codes only for IgM, IgD, and IgY [38, 41]. Taking into account the fact that both mammals and birds encode the IgA isotype, the absence of this gene in certain reptiles indicates that the IgA gene was independently lost in some reptilian lineages.

Amphibians, which represent the transitional group in the dawn of tetrapod evolution, and in particular, the frog, express five different IgH isotypes, IgM, IgD, IgX, IgY, and IgF [42] (Table **1**). Apart from these five isotypes an additional isotype, designated as IgP (π), was identified in the Iberian ribbed newt (*Pleurodeles waltl*) (Class: Amphibia; Order: Urodela) [43]. The IgF (φ) isotype contains only two constant domains and a hinge region, which covalently links the two heavy chains [42]. IgY is found in a variety of amphibians, reptiles, and birds, usually consists of four constant region domains, and is regarded as a progenitor of both mammalian IgG and IgE [44, 45]. Common biological properties and sequence homology between the IgY and IgE heavy chains suggest that IgY is the immediate ancestor of mammalian IgE, whereas the transition from IgY to IgG involved structural changes that led to the formation of a hinge region in the IgG molecules. The amphibian IgX (χ) isotype is considered an analogue of the mammalian IgA because of similarities in expression patterns and tissue distribution [44, 45]. However, somewhat different from mammalian IgA, IgX is structurally similar to avian IgA and the reptilian IgA-like molecule [40]. The mammalian IgAs therefore, may be the descendants of the amphibian IgX.

### Genomic Organization of the IgH-Encoding Loci

**Fig. (1):**
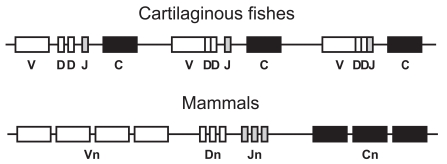
Diagrammatic representation of cluster and translocon type of immunoglobulin genomic organization with reference to the IgH loci. Cartilaginous fishes have cluster type of organization including germline-joined genes, whereas higher vertebrates have translocon type of Ig organization. C = constant gene; J = joining gene; D = diversity gene; V = variable gene; and n=variable number of genes.

In jawed vertebrates, the Ig heavy chain (IgH) encoding locus contains variable (V_H_), diversity (D_H_), joining (J_H_), and constant (C_H_) region genes. The IgH loci of the cartilaginous fishes, the earliest extant jawed vertebrates, show two exceptional characteristics as compared to all other vertebrates. Specifically, the IgH locus of cartilaginous fishes is organized as a cluster (Fig. **1**), with the most common type being a cluster composed of closely linked (V_H_-D_H_1-D_H_2-J_H_-C_H_)_n_ genes [46-50], which are dispersed throughout the genome of cartilaginous fishes [46-50]. The presence of germline-joined IgH genes is another characteristic unique to the elasmobranches. Approximately 50% of the germline IgH loci are partially (V_H_D_H_-J_H_) or fully (V_H_D_H_J_H_) joined and lack intervening sequences between the V-domain encoding genes [46-48]. The identification of germline joined Ig genes raises questions concerning their origin, evolution, and function. Accumulating evidence suggests that these sequences are the products of recombination between split genes in germ cells and most probably do not represent the primordial form of rearranging Ig genes [51, 52]. Additionally, the presence of germline joined genes may be advantageous, because these genes are no longer tied to somatic Recombination activating gene (RAG) expression and subsequent RAG-mediated rearrangement, and may not be related to the restriction of antibody diversity.

In contrast to the cartilaginous fishes, the IgH loci of bony fishes and tetrapods are organized in a locus containing multiple variable region (V_H_), joining region (J_H_) /diversity region (D_H_), genes followed by constant region (C_H_) genes (Fig. **1**). This type of organization, known as the translocon type, is conserved to all higher vertebrates. However, in some primitive bony fishes like the channel catfish (*Ictalurus punctatus*), the IgL gene organization is that of the cluster type [49, 53, 54]. The conversion from one type of organization to another, cluster versus translocon, may have represented an evolutionary advantage towards enhanced antibody diversity.

The length of the IgH loci organized as translocons varies significantly from species to species, spanning from hundreds to thousands of kilobases. The number of genes also varies extensively between different vertebrate lineages. For example, in the frog, *Xenopus tropicalis*, the IgH locus contains nearly eighty V_H_ genes followed by the five D_H_ and seven J_H_ [17, 42], whereas the IgH locus in anole lizards (*A. carolinensis*) contains more than 70 V_H_, 22 D_H_, and nine J_H_ genes [38]. The IgH locus of birds has an unusual organization as compared to other tetrapods [17, 33, 34, 37]. Specifically, the chicken IgH locus consists of a single functional V_H_ gene and 58 V_H_-pseudogenes [17, 55]. Approximately fifteen D_H_ genes and a single J_H_ are found in the chicken IgH locus [56]. In the chicken IgH-chain encoding loci (and the IgL-chain encoding loci) after rearrangement, the V(D)J region is modified extensively by gene conversion using the upstream pseudogenes as conversion donors. In humans, depending on the haplotypes, the IgH locus contains nearly 100 V_H_, 20 D_H_, and nine J_H_ genes [17, 57, 58]. More than 50% of V_H_ genes in humans are pseudogenes. Whether these V_H_ pseudogenes in humans have any role in the antibody diversity through gene conversion events is not yet precisely known. Similar variation in the gene content is also observed in the translocon-type of the IgL loci. These variations in the number of component genes suggest that in spite of the similar overall organization of the Ig-encoding loci in tetrapods, the number of component genes varies extensively and is consistent with a model of birth-and-death evolution [17, 58-61].

### Evolution of IgH Class Switching and Somatic Hypermutation

To combat a large number of antigens, a wide range of antibodies is produced by several diversifying mechanisms. During Ig gene recombination, a specialized DNA polymerase enzyme known as terminal deoxynucleotidyl transferase (TDT) introduces junctional diversity by adding N-nucleotides at the junction of V(D)J recombination; whereas B-lymphocytes upon encountering antigens in the peripheral lymphoid compartments can change the class of expressed antibody from IgM to IgG, IgA, or IgE through a recombination/deletion process termed immunoglobulin heavy chain (IgH) class switch recombination (CSR). CSR is a deletional recombination event that occurs *via *the introduction of DNA double-stranded breaks into two participating switch (S) regions, rejoining of the broken S regions to each other accompanied by deletion of all of the intervening sequences including various CH genes. A B-cell-specific protein known as activation-induced cytidine deaminase (AID) is required for the CSR activity [62]. AID is also involved in the initiation of somatic hypermutation, which introduces point mutations, and sometimes small insertions and deletions, into the variable region gene segments at a very high rate during B-cell clonal expansion.

From an evolutionary point of view, somatic hypermutation is observed in the cartilagenious fishes, whereas amphibians are the most primitive vertebrates known to use DNA recombination to switch antibody classes. Both mechanisms for antibody diversity have been maintained in every vertebrate group that has subsequently evolved [63]. Despite the expression of AID, the CSR process has not been demonstrated in bony fish and elasmobranches. However, AID from zebrafish can restore normal CSR in AID-deficient mouse B-cells, indicating that the AID functional domains required for CSR existed before the emergence of land vertebrates [64]. It is possible that components required for CSR other than AID are absent in cartilaginous and bony fishes. For example, the appropriate DNA switch regions are missing in the fish heavy chain loci. Therefore, it seems possible that the limiting step for class switching was not the evolution of a switch-capable AID protein, but rather the evolution of appropriate DNA switch regions in the translocon type configuration.

## IMMUNOGLOBULIN LIGHT CHAINS

### Evolution of Immunoglobulin Light Chain Isotypes

In humans there are two IgL isotypes, kappa (κ) and lambda (λ), which were initially recognized serologically [65]. The κ and λ denomination has been extended from humans to all other vertebrate species, most commonly by comparisons of nucleotide or amino acid sequences. These two isotypes are conserved in all jawed vertebrates (Table **1**) [49, 50]. Like humans, most mammals studied contain both κ and λ IgL chains, while analyses of IgL genes in different avian species indicated that only the λ IgL isotype is present in birds [15, 23, 59]. Use of molecular cladistic markers showed that amphibians in addition to κ and λ chains also contain genes that code for a third type, named sigma (σ) [49, 59]. The IgL isotype discrimination in lower vertebrates is problematic because the cladistic molecular markers which define κ, λ, and σ isotypes in tetrapods are not well preserved in bony and cartilaginous fishes. Nevertheless, application of these markers, as well as phylogenetic and sequence analysis suggest the presence of three isotypes in bony fishes, which are homologous to the amphibian κ, λ, and σ [50, 54]. Moreover, four IgL isotypes (σ, σ-cart, κ, and λ) have been reported in cartilaginous fishes based on phylogenetic reconstruction and the length of the CDRs [66]. The distribution of the IgL isotypes in jawed vertebrates (Table **1**) indicates that IgL isotype divergence occurred early in jawed vertebrate evolution.

### Genomic Organization of Ig Light Chain Loci

The Ig light (IgL) locus possesses variable (V_L_), joining (J_L_), and constant (C_L_) region genes. Like the IgH loci, the IgL chain encoding genes in cartilaginous fishes are present as multiple V_L_-J_L_-C_L_ clusters in the genome, with some loci being capable of rearranging while others contain fused V_L_J_L_ genes [51, 52]. Similar organization has been also identified in most bony fishes, while chondrostean fishes, like the sturgeon, have their IgL genes organized as translocons like all tetrapod species [50].

**Fig. (2):**
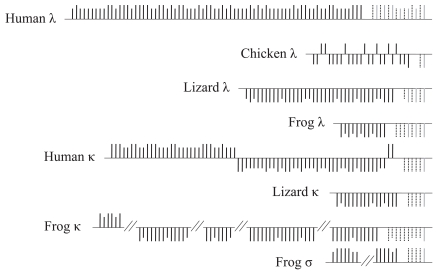
Genomic organization of immunoglobulin light chain loci in representative tetrapod species. Constant, joining and variable genes are represented by gray, dotted black, and solid black vertical lines, respectively. Long vertical lines indicate functional genes whereas short vertical lines indicate pseudogenes. Vertical lines above and below the horizontal lines indicate that the Ig genes are located on opposite strands. In the frog κ- and σ-encoding loci the variable genes are located in different scaffolds. For this reason, these two loci are represented by separate horizontal lines. The figure is not drawn to scale. See details in Das *et al*. 2008.

In most species, including tetrapods, the genes encoding the different IgL isotypes are located in different genomic regions. In the κ-encoding locus of tetrapods, multiple J_κ_ genes are present in a cluster, followed by a single C_κ_ gene. A notable exception is the κ-encoding locus of rabbits, which contains two C_κ_ genes as a result of a lineage-specific duplication [67]. In contrast, in the λ-encoding locus J_λ _and C_λ _genes occur as J_λ_–C_λ_ blocks and usually are present in multiple copies. In the λ locus, the J_λ_ gene is located within a 3-kb 5′ region upstream of the C_λ_ gene, whereas in the κ locus the J_κ_ cluster is located around 6-kb upstream of the C_κ_ gene. Birds also have unique characteristics in the organization of their IgL locus. In contrast to the organization observed in amphibians, reptiles, and mammals, birds contain only one J_λ_–C_λ_ block [15, 59]. Interestingly, the organization of the IgL-encoding loci of reptiles resembles more the organization observed in amphibians and mammals than the organization found in birds. Finally, the genomic organization of the amphibian σ-encoding locus resembles that of the κ-encoding locus, suggesting an evolutionary link between the two regions (Fig. **2**).

The numbers of the IgL chain variable genes in tetrapods vary significantly even between closely related species. For example, in rodents *V_κ_* genes are more abundant than *V_λ_* genes [59], while the *V_κ_* and *V_λ_* genes in humans are present in similar numbers. The numbers of *V_κ_* and *V_λ_* genes in platypus and opossum (non-placental mammals) also differ considerably. On the other hand, birds contain only one functional *V_λ_* gene and multiple *V_λ_* pseudogenes [15, 59]. In the anole lizard there are 16 *V_κ_* and 38 *V_λ_* genes [59, 68, 69]. In addition to the number of variable genes, the copy numbers of *IGCL*, *IGJL*, and *IGJK* genes also vary from species to species.

### Primate Specific Innovation: Novel Association between V_λ_ and microRNA Genes

**Fig. (3):**
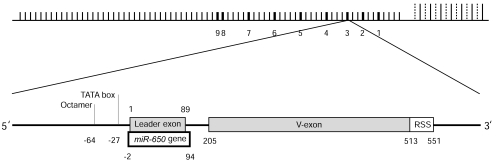
Top Panel: Genomic architecture of *miRNA-650*-bearing *V_λ_* genes in the human Ig-λ locus. The numbers the diagram indicate the positions of miR-650-bearing *V_λ_* genes. Short vertical lines indicate *V_λ_* genes, whereas long vertical lines indicate either Ig-λ constant genes (solid line) or joining genes (dotted line). Lower Panel: Schematic diagram of the overlap of *miR-650* gene and the leader exon of *V_λ_* gene is shown in a representative *miR-650*-bearing *V_λ_* gene. Numbers correspond to the position of *miR-650* gene and the positions of octamer, TATA box, leader exon, V-exon, and recombination signal sequence (RSS).

MicroRNAs (miRNAs) are single stranded small non-coding RNA molecules that regulate gene expression at the post-transcriptional level [70]. Recently, it was found that specific *V_λ_* genes contain a particular miRNA gene, known as miR-650 [71]. The *miR-650* gene (hairpin structure is 96 nt long) overlaps in the same transcription orientation with the leader exon (89 nt long) of *V_λ_* genes (Fig. **3**). The Untranslated Region (UTR) of the *V_λ_* leader exon contains the mature miRNA sequence, whereas its complementary sequence is located in protein-coding region of the leader exon (CDS). Nine miR-650-bearing *V_λ_* genes were found in humans, present in both functional and pseudogenes, and all of them belong to a specific phylogenetic group, which indicates that those *V_λ_* genes have common origin [71]. Sequence comparison and structural prediction suggested that this novel association between *miR-650* gene and the leader exon of *V_λ_* gene is a primate-specific innovation [71]. Computational analysis of the promoter region of *miR-650*-bearing *V_λ_* genes indicated that *V_λ_* and the *miR-650* genes use the same promoter region for their transcription; however these two genes are apparently transcribed independently as they are expressed in different cell types [71].

## CONCLUSIONS

The immunoglobulin, one of the major components of the jawed vertebrate’s adaptive immune system, has evolved to recognize and respond to an exceptionally diverse range of antigens. The recent availability of several vertebrate genome sequences shows a diverse and complex picture of their evolutionary dynamics. The high degree of sequence identity between specific regions of Ig genes and proteins indicates that within this multigene family, negative selection may be operating to conserve the overall folding pattern and regions responsible for recombination and cell-signaling. However, other regions of the antibodies may have been targets of diversifying selection aiming to increase diversity. Furthermore, gene copy numbers and the ratio between functional genes and pseudogenes vary greatly among species suggesting the action of a birth-and-death mode of evolution. The Ig-loci exhibit a high degree of evolutionary turnover and might be less constrained than other genomic regions for recombination, duplication, deletion, gene conversion, point mutation, and translocation. These processes facilitate the birth-and-death of individual genes in the Ig-loci. Additionally, certain IgH isotypes have independently appeared and disappeared in different vertebrate lineages, which may affect the effector functions of the antibodies and induce specialized responses in specific cell types and different species.

Like the evolution of the IgH isotypes, the evolution of Ig light chains in vertebrates is also puzzling. Four isotypes (κ, λ, σ, and σ-cart) are found in sharks, three in bony fishes and amphibians, only two (κ, and λ) in reptiles and mammals, while birds contain only on isotype (λ). This phylogenetic distribution suggests that more recently diversified vertebrate species possess lower number of different IgL chain types. This interpretation contrasts the generally accepted and well established view of increased complexity in vertebrates like mammals. The evolutionary pattern and multiplicity of the IgL types is also enigmatic, since there is no known advantage in having multiple and different IgL isotypes. Moreover, the independent evolution of antibodies composed exclusively of heavy chains, like the IgNAR in cartilaginous fishes and the heavy chain antibodies in camelids, further challenges the existing paradigms as to the immunoglobulin structure and antibody repertoire generation. Perhaps, the main advantage of multiple IgL isotypes is redundancy; if the rearrangement of genetic elements encoding one light chain isotype becomes nonfunctional, the other ones can take over.

For the generation of Ig repertoires different species use different molecular and cellular strategies but overall these strategies have evolved towards the same goal, the production of a large number of diverse Ig molecules carrying different antigen-recognizing domains. For example, unlike teleosts, the rearranged *VDJ* gene segments in the heavy chain loci of tetrapods can join to different *C* gene segments through class-switch recombination. Avian species diversify their Ig repertoires mainly by gene conversion, while B-cells in some mammalian species like sheep and cow diversify their Ig repertoire mostly by hypermutation. In spite of such diversities in the development of the Ig repertoire, the recombination mechanisms and genetic elements that promote the formation of multiple specific Igs are fairly conserved between the different vertebrate lineages. The differential use of these mechanisms suggests that evolution using the same raw materials followed different routes in the individual vertebrate lineages to generate a large number of Igs containing diverse antigen-binding regions and effector functions. The new efforts to sequence the genomes of a large number of vertebrate species using next generation sequencing technologies will enhance our understanding on the diversification processes of the immunoglobulin-encoding loci and ultimately elucidate the evolution of the adaptive immune system.

## Figures and Tables

**Table 1.** Distribution of the Immunoglobulin Heavy and Light Chain Isotypes in Vertebrates

	Cartilaginous fishes	Bony fishes	Amphibians	Reptiles	Birds	Mammals
Ig heavy chain	μ, δ, IgNAR	μ, δ, ζ/τ	μ, δ, ν, χ, π$	μ, δ, α, ν	μ, α, ν	μ, δ, α, γ, ε, °*
Ig light chain	σ-cart, σ, κ, λ	σ, κ, λ	σ, κ, λ	κ, λ	λ	κ, λ

$ IgP (ф) isotype is found in newts (*Pleurodeles waltl*) and is preferentially expressed in the larval stage.

* IgO (o) isotype is found only in platypus (*Ornithorhynchus anatinus*).
